# Geophagic Clays from Cameroon: Provenance, Metal Contamination and Health Risk Assessment

**DOI:** 10.3390/ijerph18168315

**Published:** 2021-08-05

**Authors:** Georges-Ivo Ekosse, George Elambo Nkeng, Nenita Bukalo, Olaonipekun Oyebanjo

**Affiliations:** 1 Directorate of Research and Innovation, University of Venda, Thohoyandou 0950, South Africa; ekosseg@gmail.com (G.-I.E.); nenitabukalo@gmail.com (N.B.); 2 National Advanced School of Public Works (ENSTP), Yaounde 510, Cameroon; gnkeng@yahoo.com; 3 Natural History Museum, Obafemi Awolowo University, Ile-Ife 220282, Nigeria

**Keywords:** contamination, geophagia, health risk assessment, kaolinite

## Abstract

This study assessed the mineralogical and geochemical characteristics of geophagic clays sold in some markets in Cameroon to ascertain their provenance, contamination status and human health risk. To achieve this, 40 samples from 13 markets in Cameroon were purchased and analysed using X-ray diffractometry, X-ray fluorescence and laser ablation inductively coupled plasma mass spectrometry for their mineralogy and geochemistry, respectively. The geophagic clays were dominantly made up of kaolinite and quartz. Their chemistry was dominated by SiO_2_, Al_2_O_3_ and LOI with means of 48.76 wt%, 32.12 wt% and 13.93 wt%, respectively. The major, trace and rare earth elements data showed that these geophagic clays were predominantly derived from felsic rocks. The contamination assessment indicated no enrichment of metals from anthropogenic sources, except for Zn in samples from Acacia, Madagascar and Mfoudi markets. The index of geo-accumulation indicated no contamination to moderate contamination of the clays. The non-carcinogenic index values for Fe, Co, Cr, Cu, Ni, Pb and Zn were generally less than 1, suggesting no non-carcinogenic risk exposure to children and adults consuming the geophagic clays from these metals. The carcinogenic risk index (TCR) for Ni and Cr were above 10^−6^, which implies that children and adults are vulnerable to minimal carcinogenic health risk. The TCR values from Ni posed the highest risk, especially to children consuming clays from some markets.

## 1. Introduction

Geophagia, the deliberate consumption of soil by humans and animals, has been reported to occur in several parts of the world [1,2,3,4,5]. Although the practice has been traced back to Hippocrates in 400 BC, it is most common in African countries [6] and in African communities living in the West [7,8,9]. Various reasons have been advanced for the consumption of clays. About 46% to 73% of pregnant/nursing women practice geophagy to treat nausea, vomiting, abdominal pain and other pregnancy-related malaise [10,11]. It is believed that pregnant women may consume on average 20 g/day of geophagic material [12]. Other reasons include nutrient supplementation, detoxification and alleviation of gastrointestinal disorders and cravings, as well as cultural and religious beliefs [13], with nutrient supplementation being the most advanced reason for practicing geophagia.

In sub-Saharan Africa, clay used for geophagia is usually mined in large quantities for distribution for sale in markets and without any prior treatment before consumption. This clay is predominantly made up of kaolin. Kaolin could either be primary or secondary based on its genesis. Primary kaolins form in situ and they could either be hypogene (resulting from hydrothermal activities) or supergene (formed from the weathering of alumino-silicate rocks) [14]. However, secondary or sedimentary kaolins are those that formed elsewhere, then were transported and deposited in a different location [15]. Sedimentary clays are believed to be more refined, with low concentrations of elemental impurities, and they are usually preferred for geophagia because of their potential medicinal and nutritional values [16].

Based on their provenance, clays could have varied mineralogical and geochemical characteristics, which may constitute a health risk for human beings practicing geophagia [17]. When ingested or inhaled at high levels over a long period of time, trace elements are toxic to humans because most of them are neurotoxic, immunotoxic, mutagenic, teratogenic or carcinogenic agents [18,19]. They could cause brain damage, impaired red blood cells and kidneys, dysfunctional labour to pregnant women, maternal deaths and stillborns [20,21]. For instance, acute lead (Pb) exposure could cause damage in the human central nervous system, resulting in dysfunction of some organs, such as the brain, kidney, liver and heart [22]. About 20%–70% of ingested Pb is absorbed by the human body [23]. Even at low levels, Pb could affect brain development in infants [20]. Exposure to high levels of cadmium (Cd) has been associated with an increased risk of cardiovascular disease and coronary heart disease [24]. Other elements such as iron (Fe), arsenic (As) and copper (Cu) have also been associated to hepatic failure, arthritis, renal dysfunction, nausea, and diarrhoea [25].

Several studies have been carried out on geophagic clays in Cameroon [13,26,27,28,29,30,31,32,33]. In 2002, the European Commission alerted the Cameroonian Ministry of Public Health [27] about abnormally high amount of lead (100 times higher than the maximum permissible level) in geophagic kaolin carried from Cameroon to Europe. In 2016, the study by Frazzoli et al. [27] indicated that geophagic clays sold in markets in Cameroon contained high concentrations of lead, cadmium and mercury. Kenne Kalguem et al. [32] also noted that geophagic clays from Sabga (Northwest Cameroon) contain high radioactive (Th, U), carcinogenic (Cr, Cu, Pb, Ni) and teratogenic (Cu, Zn, Pb) elements. Frazzoli et al. [27] recommended that provenance studies of geophagic clays in Cameroon be carried out to establish environmental characteristics of these clays. Despite the growing health concerns regarding the chemical composition of geophagic clays in Cameroon, there has been no assessment of the non-carcinogenic and carcinogenic health risks associated with trace metals present in these clays. Hence, the aims of this study were to (i) determine the provenance of geophagic clays sold in some markets in Cameroon and (ii) carry out metal contamination and health risk assessment of selected trace metals’ intake through geophagia.

## 2. Materials and Methods

### 2.1. Materials

**Figure 1:**
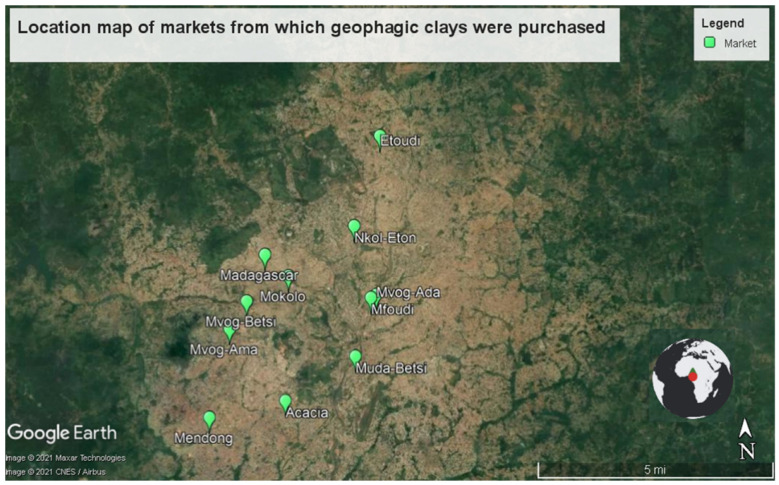
Location map of markets from which geophagic clays were purchased.

In this study, 40 geophagic clay samples were randomly purchased from 13 markets in Cameroon, predominantly in Yaounde (the capital of Cameroon), except for Balengou and Bokwango (Figure 1). The selection of the samples was based on the different varieties found in the markets. The number of samples per market, the location of the markets and the sample codes are shown in Table 1.

### 2.2. Laboratory Analyses

The purchased geophagic samples were air dried at room temperature. Then, they were gently crushed using a mortar and pestle. The samples were then sieved through a 2 mm sieve to remove plant roots in the samples. The homogenised <2 mm samples were taken as the bulk and used for analyses [34]. The mineralogical characteristics of the geophagic clays were determined by X-ray diffractometry (XRD) at XRD Analytical and Consulting at Johannesburg (South Africa). The samples were initially scanned using a backloading preparation method to reduce the effect of preferred orientation [35]. This method consists in loading the sample from the back of the sample holder and removing the excess sample with a sharp edge [36]. Diffractograms were obtain using a Malvern Panalytical Aeris diffractometer with PIXcel detector and fixed slits with an Fe-filtered cobalt source (λ = 1.789 Å) and an alpha filter (CoKα). Each sample was scanned from 5° 2θ to 80° 2θ at a rate of 2° per minute, and results reported up to 40° 2θ. The mineral phases were identified by search/match function using X’Pert Highscore plus software, with the Inorganic Crystal Structure Database (ICSD). The mineral quantification in weight % (wt %) was estimated using the Rietveld method.

The geochemical analyses were carried out at the Central Analytical Facility at Stellenbosch University (South Africa). Major oxides’ concentrations of the geophagic clays were determined by X-ray fluorescence spectrometry (XRF) using PANalytical Axios Wavelength Dispersive spectrometer. The spectrometer was fitted with a Rh tube (3 kW). The standards analysed with the samples were as follows: BE-N, JB-1, BHVO-1, JG-1, HUSG-1, WITS-G and NIM-G [37]. Loss on ignition (LOI) was determined as the weight loss or gain of each sample after heating overnight at 1000 °C.

Thirty-four trace elements’ concentrations of the geophagic clays were determined using laser ablation inductively coupled plasma spectrometry (LA-ICP-MS). The instrument used was an Agilent 7700. A laser was used to vaporise the surface of the solid sample, while the vapour, and any particles, were then transported by the carrier gas flow to the ICP-MS. Ablation was performed on pressed pellets of milled sample powder in He at a flow rate of 0.40 L/min, then mixed with Ar (0.9 L/min) and N (0.002 L/min) just before introduction into the ICP plasma. Two spots of 104 µm each were ablated on each sample using a frequency of 8 Hz and 3.5 mJ/cm^2^ energy. Quality control standards used were BHVO and BCR glass [38] and BHVO and BCR powder [39]. The calibration standard (NIST 610) was run after every 15–20 samples using standard sample bracketing. For quality assurance, two replicate measurements were carried out on each sample. The instrument’s detection limits of the analysed trace elements are shown in Table S1.

### 2.3. Trace Metal Contamination Assessment

The contamination assessment of Vanadium (V), chromium (Cr), cobalt (Co), nickel (Ni), copper (Cu), zinc (Zn), iron (Fe) and lead (Pb) was done using the enrichment factor (EF) and the geo-accumulation index (*Igeo*). Vanadium, Cr, Co, Ni, Cu, Zn and Pb are widely utilised to infer pollutants because they are considered as toxic elements and are easily mobilised by human activity [40,41]. Iron was selected because geophagists generally believe that the Fe in clays helps prevent iron deficiency anaemia [16]. The EF is usually used to differentiate between the natural and anthropogenic source of an element, using its natural background levels in the environment. A high EF value of an element is indicative of enrichment of that element in the environment as a result of anthropogenic activities. The EF is calculated as in Equation (1) [42]:

(1) $$\mathrm{EF}={\left(M_{c}/M_{r}\right)}_{\mathrm{sample}}/{\left(M_{c}/M_{r}\right)}_{\mathrm{background}}$$

where M_c_ is the concentration of the metal in the geophagic clays and M_r_ is the concentration of the reference element. According to Salati and Moore [42], Ti is one of the immobile elements that could be used as reference element. Hence, it was used in this study. Moreover, Ti did not show significant variation in the dataset. The upper continental crust (UCC) values were used as the background [43].

The assessment of the trace metal accumulation in the studied geophagic clays was carried out using the geo-accumulation index (*Igeo*) as given in Equation (2) [44]:

(2) $$Igeo=\log_{2}\left(\frac{C_{i}}{1.5\times \mathrm{GBV}}\right)$$

where C_i_ is the measured concentration of metal i and GBV is the geochemical background value (UCC value) of the same metal.

The interpretation of the EF and *Igeo* values is given in Table S2.

### 2.4. Human Health Risk Assessment

The human health risk was assessed by evaluating the non-carcinogenic and carcinogenic risk. Individuals are exposed to soil trace metals in through three main pathways [45,46]: (i) direct oral ingestion (as it is the case with geophagia), (ii) inhalation of suspended soil particles and (iii) dermal absorption through exposed skin. The average daily intakes (ADIs) for non-carcinogens and carcinogens through each exposure pathway and the sum of all pathways were calculated using Equations (3) to (6) [46,47,48].

(3) $${\mathrm{ADI}}_{\mathrm{inh}}=\frac{\mathrm{Ci}\times {\mathrm{IR}}_{\mathrm{air}}\times \mathrm{EF}\times \mathrm{ED}}{\mathrm{PEF}\times \mathrm{BW}\times \mathrm{AT}}$$

(4) $${\mathrm{ADI}}_{\mathrm{dermal}}=\frac{\mathrm{Ci}\times \mathrm{SA}\times \mathrm{AF}\times \mathrm{ABS}\times \mathrm{EF}\times \mathrm{ED}}{\mathrm{BW}\times \mathrm{AT}}\times \mathrm{CF}$$

(5) $${\mathrm{ADI}}_{\mathrm{ing}}=\frac{\mathrm{Ci}\times {\mathrm{IR}}_{\mathrm{soil}}\times \mathrm{EF}\times \mathrm{ED}}{\mathrm{BW}\times \mathrm{AT}}\times \mathrm{CF}$$

(6) $${\mathrm{ADI}}_{T}={\mathrm{ADI}}_{\mathrm{inh}}+{\mathrm{ADI}}_{\mathrm{dermal}}+{\mathrm{ADI}}_{\mathrm{ing}}$$

The hazard quotient (HQ) and the hazard index (HI) were used to evaluate the non-carcinogenic risk [46]. They were computed as shown in Equations (7) and (8) [47], as follows:

(7) $$\mathrm{HQ}=\frac{\mathrm{ADI}}{\mathrm{RfD}}$$

(8) $$\mathrm{HI}=\sum {\mathrm{HQ}}_{i}=\sum \frac{{\mathrm{ADI}}_{i}}{{\mathrm{RfD}}_{i}}$$

where RfD is the reference dose (mg/kg/day) of each trace metal. According to [47]:

- If the HI < 1, no risk of non- carcinogenic effects is believed to occur.
- If the HI value > 1, there is probability of potential non-carcinogenic effects on humans.

The carcinogenic health risk for an individual heavy metal over a lifetime (CR) and the total carcinogenic health risk (TCR) of all selected trace metals were computed according to Equations (9) and (10), respectively [46,47].

(9) CR=ADI×SF

where SF is the slope factor.

(10) TCR=∑CR

Values of CR lower than 10^−6^ are considered insignificant, values of risk between 10^−6^ and 10^−4^ are considered as excess cancer risks and CR values greater than 10^−4^ are considered harmful to humans [48,49]. The definitions and units of parameters and values used in Equations (1)–(8) are listed in Table S3, and the reference doses (RfD) for non-carcinogenic trace metals and slope factors (SF) for carcinogenic trace metals are found in Table S4.

## 3. Results

### 3.1. Mineralogy

**Figure 2:**
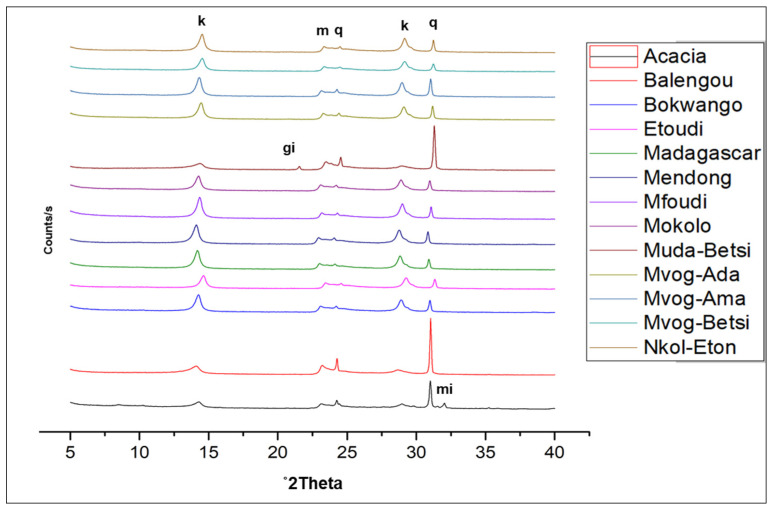
X-ray diffractograms of the studied geophagic clays from the various markets with the highest kaolinite contents showing kaolinite (k), quartz (q), muscovite (m), gibbsite (gi) and microcline (mi).

**Figure 3:**
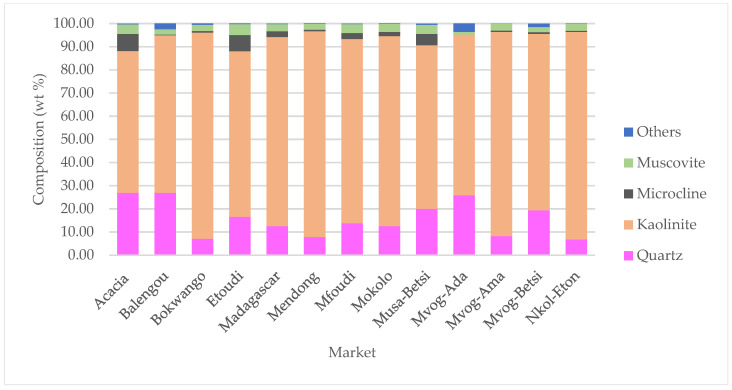
Abundances of mineral phases present in geophagic clays sold in markets in Cameroon. “Others” represent the sum of sepiolite, hematite, gibbsite and goethite.

In Figure 2, the X-ray diffractograms of geophagic clays from the various markets with the highest kaolinite contents are plotted, and Figure 3 shows the abundances of the different mineral phases present in the studied geophagic clays sold in Cameroon markets. These were dominantly made up of kaolinite, with means ranging from 61.20 wt% (Acacia geophagic clays) to 89.63 wt% (Nkol-Eton geophagic clays). Quartz was the second most abundant mineral, with means ranging from 6.75 wt% (Nkol-Eton geophagic clays) to 26.93 wt% (Balengou geophagic clays). Microcline and muscovite were minor mineral phases found in the geophagic clays, having means of 2.30 and 3.10 wt%, respectively. In addition, trace amounts of sepiolite, hematite, gibbsite and goethite were determined in some of the samples.

### 3.2. Geochemistry

**Figure 4:**
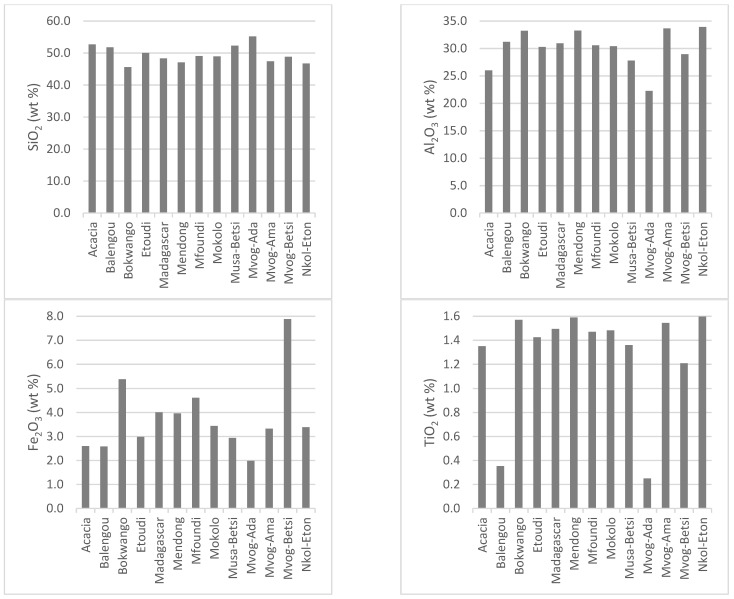

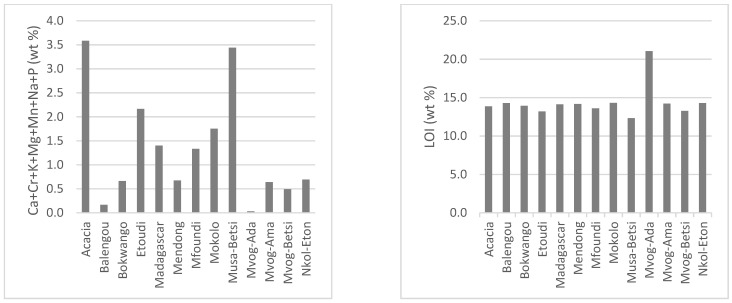
Concentration of major oxides, minor oxides and LOI in the studied geophagic clays.

Being mainly made up of kaolinite, the chemistry of studied geophagic clays was predominantly made up of SiO_2_ (mean of 48.76 wt%), Al_2_O_3_ (mean of 31.12 wt%) and LOI (mean of 13.93 wt%). Iron (Fe_2_O_3_) was the main impurity, varying between 2.58 wt% in the Balengou samples and 7.88 wt% in the Mvog-Betsi samples. Concentrations of TiO_2_, which was also a main impurity in kaolins, varied between 0.35 wt% (Balengou) and 1.60 wt% (Nkol-Eton). Concentrations of other oxides (CaO, Cr_2_O_3_, K_2_O, MgO, MnO, Na_2_O and P_2_O_5_) were generally less than 1 wt%. The concentrations of major oxides, minor oxides and LOI in the studied geophagic clays are shown in Figure 4. In all samples, the K_2_O/Al_2_O_3_ ratio was less than 0.1.

**Figure 5:**
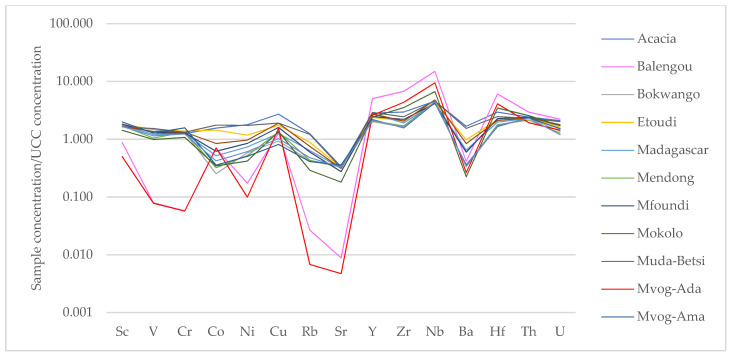
Spider diagrams showing upper continental crust (UCC)-normalised trace elements in the studied geophagic clays.

The trace and rare earth elements concentrations are presented in Tables S5 and S6, respectively. When compared to UCC values of trace elements [43], the Muda-Betsi and Acacia geophagic clays showed similar trends, i.e., slight enrichment of trace elements, except for a depletion of Sr (Figure 5). These clays were slightly more enriched in high field strength elements (HFSEs—Y, Zr, Nb and Hf). The Balengou and Mvog-Ada geophagic clays also showed similar trends of trace elements relative to UCC. They were enriched in all HFSEs, depleted in large ion lithophile elements (LILEs—Rb, Ba, Sr, Th and U), though enriched in Th and U, and depleted in transition trace elements (TTEs—V, Co, Cu, Ni and Sc), except in Cu.

**Figure 6:**
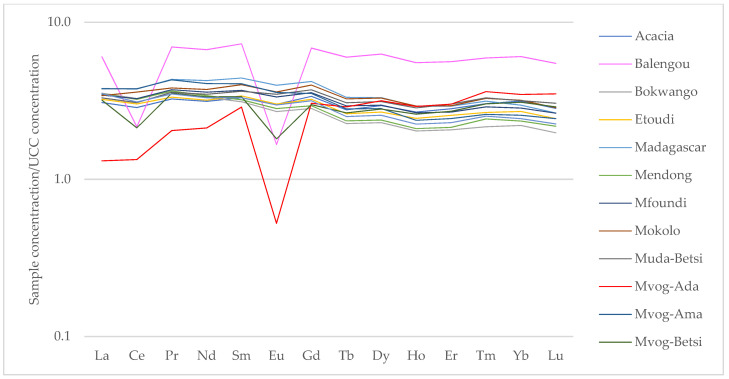
Spider diagrams showing upper continental crust (UCC)-normalised rare earth elements in the studied geophagic clays.

The UCC-normalised rare earth elements (REEs) showed enrichment in all the geophagic clays, except the Mvog-Ada clays, which were depleted in Eu (Figure 6). All samples also portrayed a negative europium anomaly (Eu/Eu*) ranging from 0.18 (Mvog-Ada) to 0.95 (Muda-Betsi).

## 4. Discussion

### 4.1. Provenance of Geophagic Clays

#### 4.1.1. Weathering and Source Rocks of Geophagic Clays

The geochemistry of clays has commonly been used to determine their provenance because clays retain the geochemical signatures of their source rocks. Major oxides and trace elements of clay minerals could be a best indicator for their compositional variability, thereby giving insights on their provenance, as well as environmental conditions prevailing during diagenesis [50]. The chemical index of alteration (CIA) and the index of compositional variability (ICV) were respectively used to determine the intensity of weathering and the maturity of the studied geophagic clays. The ICV was used to measure the abundance of Al_2_O_3_ relative to the other major oxides in the kaolins. These indices were calculated based on equations developed by Nesbitt and Young [51] (Equation (11)) and Cox et al. [50] (Equation (12)).

(11) $$\mathrm{CIA}=\left[{\mathrm{Al}}_{2}O_{3}/\left({\mathrm{Al}}_{2}O_{3}+{\mathrm{CaO}}^{*}+{\mathrm{Na}}_{2}O+K_{2}O\right)\right]\times 100$$

(12) $$\mathrm{ICV}=\left(\mathrm{CaO}+K_{2}O+{\mathrm{Na}}_{2}O+{\mathrm{Fe}}_{2}O_{3}\left(t\right)+\mathrm{MgO}+\mathrm{MnO}+{\mathrm{Ti}}_{2}O\right)/{\mathrm{Al}}_{2}O_{3}$$

For the calculation of these indices, all values are in molar contents, with CaO* being the amount of CaO incorporated in the silicate fraction of the rock, and SiO_2_ is excluded to eliminate the challenges of quartz dilution.

**Figure 7:**
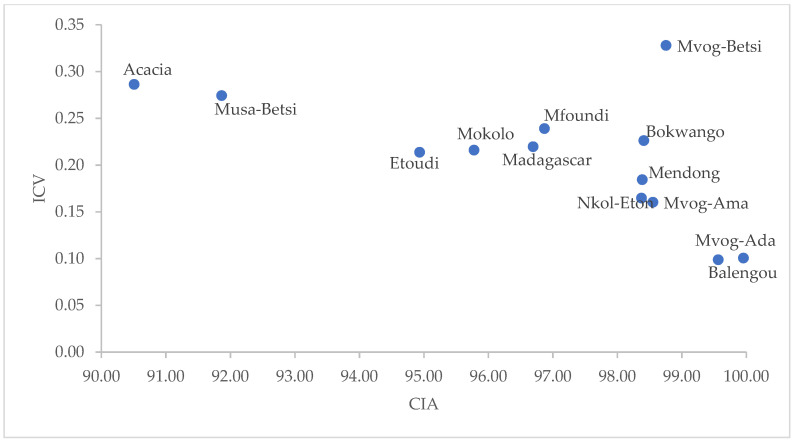
Plot of ICV versus CIA.

In Figure 7, the CIA of the geophagic clays indicated extreme silicate weathering (CIA > 80). High CIA values indicate increased weathering of sediments because of high rainfall [52]. Extreme silicate weathering causes a higher mobility of Na, K and Ca, leaving the immobile Al in sediments [53]. However, as weathering progresses, ICV decreases due to the conversion of feldspars to Al-bearing clays, such as kaolins. Having a low ICV (<0.35) and being dominantly made up of kaolinite, the geophagic clays were compositionally mature, which might have been deposited in tectonically quiescent or cratonic environments [50,54].

**Figure 8:**
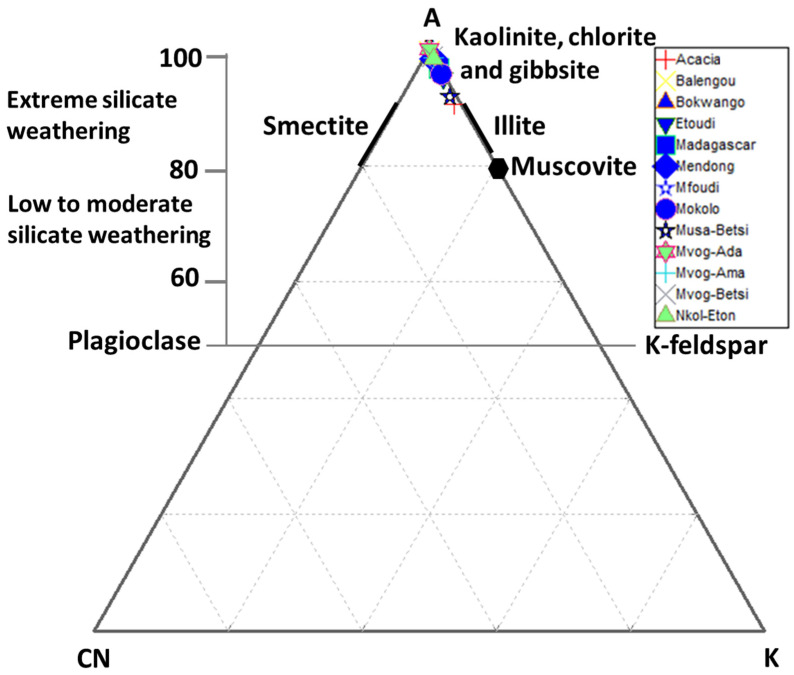
Ternary plot of A (Al_2_O_3_) − CN (CaO* + Na_2_O) – K (K_2_O) combined with CIA.

The A-CN-K ternary plot (Figure 8) was used to evaluate the geochemical alteration of studied geophagic clays [55,56]. This plot identifies the differentiation of compositional changes associated with chemical weathering and/or source rock composition [57]. The studied geophagic clays plotted close to the Al_2_O_3_, confirming the high degree of weathering and a mineralogy dominated by kaolinite (Figure 8).

**Figure 9:**
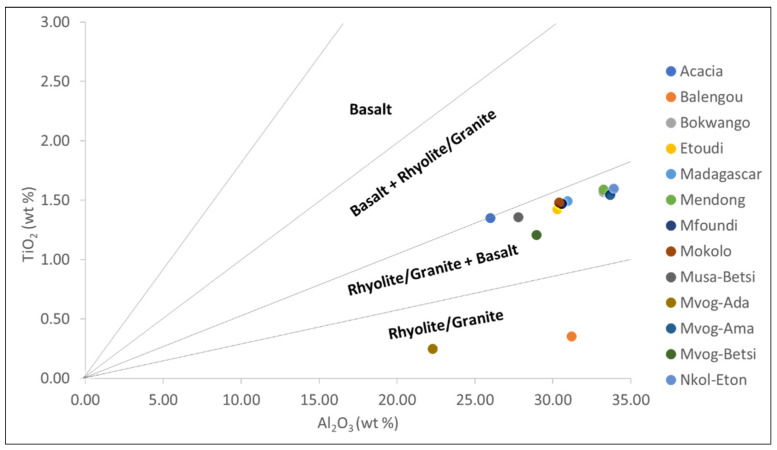
TiO_2_-Al_2_O_3_ binary plot of bulk samples (fields from Ekosse [59]). Reprinted with permission from ref. 51213800615102. Copyright 2001 Elsevier.

Girty et al. [58] used the Al_2_O_3_/TiO_2_ ratio to discriminate between mafic and felsic rocks: the Al_2_O_3_/TiO_2_ ratio is <14 in mafic rocks, between 19 and 28 in intermediate rocks, and >28 in felsic rocks. The Al_2_O_3_/TiO_2_ ratios per market generally varied between 19.25 and 27.25, suggesting intermediate source rocks (Table 2). Only the Balengou and Mvog-Ada geophagic clays had an Al_2_O_3_/TiO_2_ ratio of 88.26 and 89.08, respectively, suggesting a felsic source. Conversely, in the TiO_2_-Al_2_O_3_ binary plot, the Balengou and Mvog-Ada geophagic clays were plotted in the rhyolite/granite field, while the other clays were plotted in the rhyolite/granite + basalt field (Figure 9).

**Figure 10:**
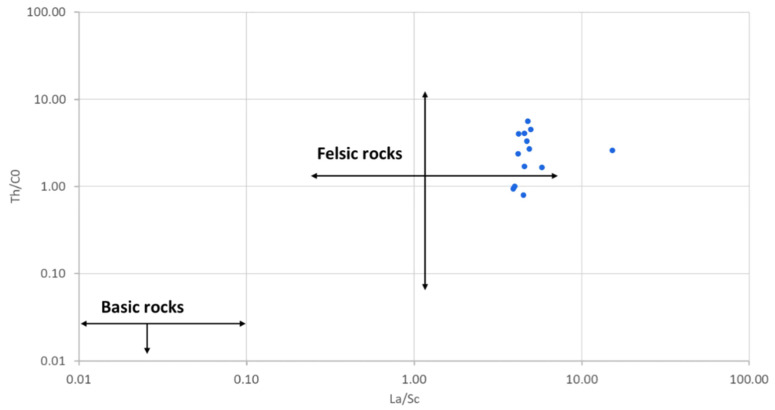
Th/Co vs. La/Sc showing the source rocks of studied geophagic clays. Fields from López et al. [60]. Reprinted with permission from ref. 5121390273963. Copyright 2005 Elsevier.

The reduced mobility of trace elements (including rare earth elements) during geological processes enables them to preserve the chemical signatures of their parent rocks during erosion and sedimentation [60], giving them distinct geochemical behaviour in natural systems [61]. This makes them useful indicators of provenance of sediments [62]. Thorium (Th), Sc, Cr, Ni, La and Co, which are among the least soluble, are the widely used for provenance studies, because they are transported almost exclusively in the terrigenous component of a sediment and, therefore, reflect the chemistry of their source rocks [60]. The La/Sc, Th/Sc, Th/Co and Th/Cr were used to determine the provenance of the studied geophagic clays. They ranged between 3.90 and 15.20, 0.89 and 2.89, 0.80 and 5.62 and 0.17 and 5.86 for La/Sc, Th/Sc, Th/Co and Th/Cr, respectively (Table 2). These ranges indicated that the geophagic clays were derived from felsic parent rocks [63]. Felsic rocks are richer in Th and La; whereas the mafic rocks are richer in Co, Sc, Ni and Cr [57]. The Th/Co vs. La/Sc plot (Figure 10) was used to discriminate between felsic and basic source rocks [60]. It showed that the geophagic clays from Cameroon markets had a felsic source.

#### 4.1.2. Sediment Sorting and Recycling

**Figure 11:**
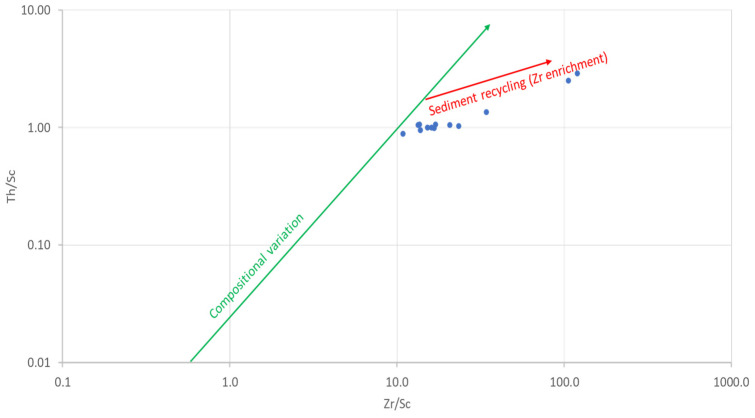
Th/Sc versus Zr/Sc plot showing sedimentary recycling in studied geophagic clays. Fields from McLennan et al. [67]. Reprinted with permission from ref. 1138320-1. Copyright 1993 Geological Society of America.

The amounts of Zr and Th are controlled by hydraulic sorting in sedimentary rocks [64]. Thorium is incompatible, whereas Sc is compatible in igneous systems [65]. Zirconium is strongly enriched in zircon, whereas Sc is not enriched but generally preserves a signature of the provenance. The Th/Sc and Zr/Sc ratios can be used as proxies for igneous chemical differentiation processes and for zircon enrichment, respectively [66,67]. Therefore, the Th/Sc vs. Zr/Sc bivariate plot has been used to illustrate hydraulic sorting and sedimentary recycling [67]. The first trend is that of compositional variability, showing the normal igneous differentiation trend, which does not involve zircon enrichment. The second trend is that in which there is an enrichment of zircon due to sedimentary sorting or recycling [64]. The Th/Sc vs. Zr/Sc of the studied geophagic clays plotted along the second, which shows sedimentary sorting and recycling (second cycle sediments) (Figure 11).

### 4.2. Contamination Assessment

#### 4.2.1. Enrichment Factor (EF)

**Figure 12:**
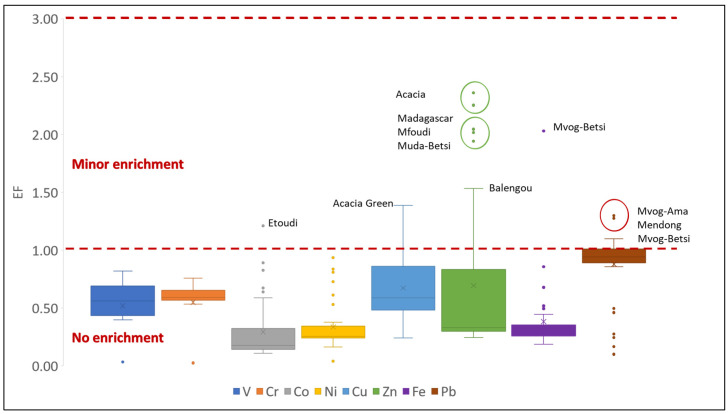
Enrichment factor of selected metals in studied geophagic clays.

The EF is usually used to differentiate between natural and anthropogenic source of an element, using its natural background levels in the environment. In this study, Ti was used as the reference element because it is immobile [42], and it did not show significant variation within the dataset. A high EF value of an element is indicative of enrichment of that element in the environment because of anthropogenic activities. In general, EF values < 2 indicate a lithogenic origin, whereas EF values > 2 indicate an anthropogenic source [68]. Mean EF values of selected metals in studied geophagic clays were below 1, varying between 0.29 (Ni) and 0.88 (Pb). This indicates that most of the geophagic clays sold in markets in Cameroon show no enrichment of metals from anthropogenic sources [69]. Zn was the only metal having more than one EF value greater than two. These were found in geophagic clays from Acacia (2.36 and 2.25), Madagascar (2.04) and Mfoudi (2.02) markets (Figure 12). Possible anthropogenic sources of Zn include motor oil, grease, batteries, pesticides, phosphate fertilisers, sewage sludges, transmission fluid, under coating, incineration and wood combustion [70]. Excessive consumption of Zn has been linked to abdominal cramps and interference with Cu metabolism in the body, especially in young males and adolescent females [71,72].

#### 4.2.2. Index of Geo-Accumulation (*Igeo*)

**Figure 13:**
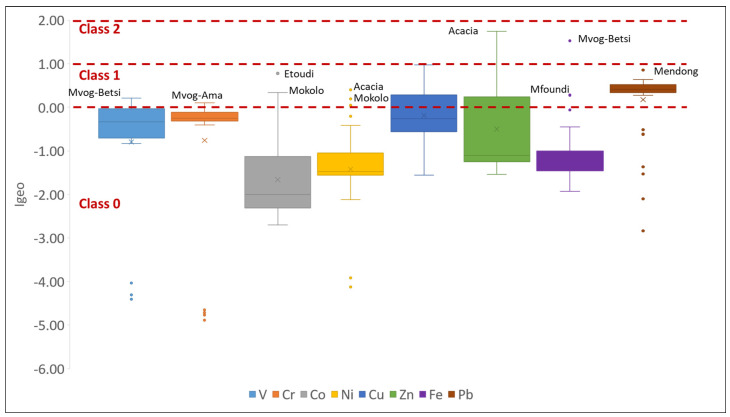
The *Igeo* of selected metals in the studied geophagic clays.

The mean values of the *Igeo* of selected metals in studies of geophagic clays in Cameroon varied from 1.66 (Co) < −1.42 (Ni) < −1.09 (Fe) < 0.80 (V) < −0.76 (Cr) < −0.50 (Zn) < −0.19 (Cu) < 0.18 (Pb). The *Igeo* values were generally less than 0 (Class 0), suggesting no contamination of the geophagic clays (Figure 13). However, some samples were plotted in Class 1 (0 < *Igeo* < 1) and Class 2 (1 < *Igeo* < 2) of the *Igeo* classification, suggesting no contamination to moderate contamination. Some of these samples include clays from Mvog-Betsi, which were plotted in Class 1 for V and Class 2 for Fe; Mokolo clays, which were plotted in Class 1 for Co and Ni; and Acacia clays, which were plotted in Class 1 for Ni and Class 2 for Zn. Moreover, the *Igeo* of Pb was generally plotted in Class 1, indicating that they are uncontaminated to moderately contaminated. 

### 4.3. Health Risk Assessment

**Figure 14:**
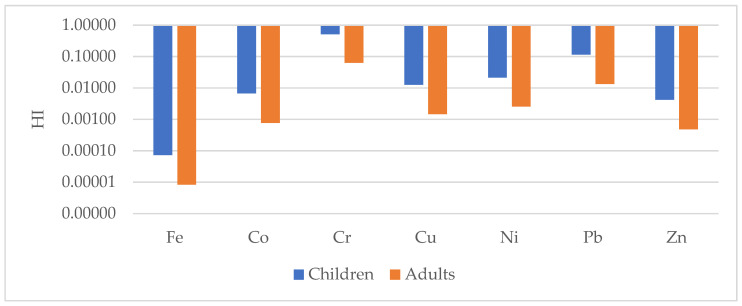
Non-carcinogenic risk hazard index (HI) for children and adults for selected metals in the studied geophagic clays in Cameroon.

**Figure 15:**
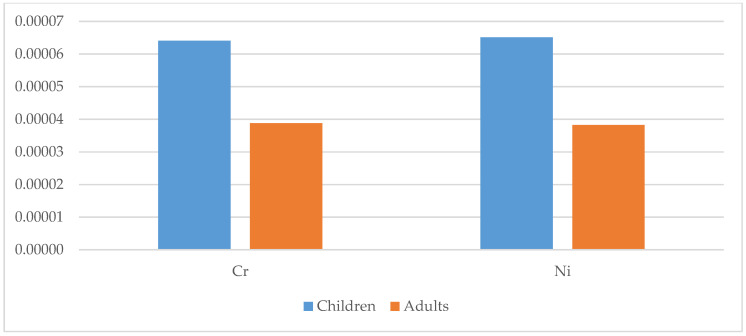
Total carcinogenic risk (TCR) for children and adults for selected metals in the studied geophagic clays in Cameroon.

The non-carcinogenic (HI) and total carcinogenic risks (TCR) through ingestion, inhalation and dermal pathways for children and adults consuming the studied geophagic clays sold in Cameroon markets are shown in Figure 14 and Figure 15 and Table 3. The following order was observed in the exposure assessment results: HQ_ingestion_ > HQ_dermal_ > HQ_inhalation_. This shows that ingestion is the main pathway from which children and adults can get exposed to the selected metals. This is of great concern because geophagists directly ingest these clays without any prior treatment. The HI values were all less than 1, with means of 7.31 × 10^−5^, 6.73 × 10^−3^, 5.10 × 10^−1^, 1.2 × 10^−2^, 2.12 × 10^−2^, 1.15 × 10^−1^ and 4.16 × 10^−3^ for Fe, Co, Cr, Cu, Ni, Pb and Zn, respectively for children; and 8.3 × 10^−6^, 7.69 × 10^−4^, 6.26 × 10^−2^, 1.45 × 10^−3^, 2.56 × 10^−3^, 1.33 × 10^−2^ and 4.79 × 10^−4^ for Fe, Co, Cr, Cu, Ni, Pb and Zn, respectively, for adults. Hence, exposure to these metals would not cause a health risk to geophagists [46,48,73]. These data agree with findings by Nkansah et al. [22], who reported HI values in geophagic white clays from Kumasi Metropolis (Ghana) to be less than 1. The same trend was also observed in geophagic kaolins from Eastern Dahomey and the Niger Delta Basins in Nigeria [25] and those studied by Lar et al. [12] in the same place. However, unlike these studies, Kortei et al. [74] showed that geophagic clays consumed by pregnant women at Ho in Ghana had HI values greater 1.

The carcinogenic health risk was determined for Cr, Ni and Pb based on their TCR values. The TCR values of Pb were all below 10^−6^. All TCR values of Cr and Ni for both children and adults were above 10^−6^, with means of 6.41 × 10^−5^ (Cr) and 5.51 × 10^−5^ (Ni) for children, and 3.88 × 10^−5^ (Cr) and 3.83 × 10^−5^ (Ni) for adults. Hence, this suggests that there was a minimal carcinogenic health risk for the population. However, the TCR values of Ni in geophagic clays from Acacia, Etoudi, Madagascar, Mokolo and Muda-Betsi markets were greater than 10^−4^, suggesting that these clays could potentially be harmful to geophagists, especially to children (Figure 13). These results were consistent with findings by Oyebanjo et al. [25], which showed that children consuming geophagic kaolinitic clays from Eastern Dahomey and the Niger Delta Basins (Nigeria) had more of a health risk than the adults.

## 5. Conclusions

The provenance, contamination status and human health risk of geophagic clays sold in selected markets in Cameroon were determined using their mineralogy and geochemistry. Based on the mineral phases present in the clays and their chemistry (major and trace elements), the geophagic clays were derived from felsic parent rocks. These clays are believed to be second cycle sediments, which underwent extreme silicate weathering in a tectonically calm environment. The enrichment factor used to assess the contamination status generally showed no enrichment of Fe, Co, Cr, Cu, Ni, Pb and Zn from anthropogenic sources, indicating a geogenic source for these metals. These metals also pose no non-carcinogenic risk to children and adults consuming these clays. However, a minimal health risk was determined for Ni and Cr in children and adults. Moreover, children are more likely to have potential harmful health risks from the trace metals in the studied geophagic clays, with Ni being the main metals of concern for both children and adults. It is therefore recommended that these geophagic clays be beneficiated before being sold in markets.

## Figures and Tables

**Table 1:** Number of geophagic clay samples per market.

Market	Coordinates	Sample Codes	Number of Samples
Acacia	3°50′7.58″ N, 11°29′50.68″ E	S1–S2	2
Balengou	5°5′60.00″ N, 10°25′60.00″ E	S3–S5	3
Bokwango	4°13′4.00″ N, 9°10′24.00″ E	S6–S8	3
Etoudi	3°54′55.61″ N, 11°31′45.33″ E	S9–S10	2
Madagascar	3°52′49.13″ N, 11°29′33.56″ E	S11–S14	4
Mendong	3°49′51.99″ N, 11°28′25.93″ E	S15–S20	6
Mfoudi	3°51′57.86″ N, 11°31′29.24″ E	S21–S24	4
Mokolo	3°52′24.77″ N, 11°29′58.80″ E	S25–S28	4
Muda-Betsi	3°50′53.88″ N, 11°31′9.81″ E	S29	1
Mvog-Ada	3°52′0.80″ N, 11°31′35.20″ E	S30	1
Mvog-Ama	3°51′28.60″ N, 11°28′51.66″ E	S31–S32	2
Mvog-Betsi	3°51′58.68″ N, 11°29′11.59″ E	S33–S36	4
Nkol-Eton	3°53′17.43″ N, 11°31′13.35″ E	S37–S40	4
Total			40

**Table 2:** Average elemental ratios in studied geophagic clays from Cameroon compared with ratios in felsic and mafic rocks.

	Al_2_O_3_/TiO_2_	La/Sc	Th/Sc	Th/Co	Th/Cr	Zr/Sc
Acacia	19.25	3.90	1.03	0.94	0.22	23.31
Balengou	88.26	15.20	2.51	2.59	5.86	106.02
Bokwango	21.17	4.77	1.07	5.62	0.20	13.61
Etoudi	21.24	3.97	1.00	1.01	0.20	16.00
Madagascar	20.69	4.85	1.00	2.71	0.21	15.23
Mendong	20.91	4.56	0.95	4.07	0.19	13.73
Mfoudi	20.79	4.17	0.99	2.38	0.21	16.66
Mokolo	20.50	4.54	1.06	1.69	0.20	16.97
Musa-Betsi	20.43	4.47	1.06	0.80	0.20	20.62
Mvog-Ada	89.08	5.80	2.89	1.65	3.85	119.74
Mvog-Ama	21.79	4.19	0.89	4.03	0.17	10.85
Mvog-Betsi	23.98	4.97	1.35	4.51	0.27	34.10
Nkol-Eton	21.22	4.70	1.05	3.32	0.21	13.36
Minimum	19.25	3.90	0.89	0.80	0.17	10.85
Maximum	89.08	15.20	2.89	5.62	5.86	119.74
Mean	31.49	5.39	1.30	2.72	0.92	32.32
Felsic rocks *	>28	0.70–27.70	0.64–18.1	0.3–7.5	0.067–4.0	
Mafic rocks *	<14	0.40–1.10	0.05–0.4	NA	0.002–0.045	

* Values from Cullers [63]. Reprinted with permission from ref. 5121390047797. Copyright 2000 Elsevier.

**Table 3:** Non-carcinogenic and carcinogenic risks through ingestion, inhalation and dermal pathways for children and adults based on selected trace metals in geophagic clays from Cameroon.

Non-Carcinogenic	Children				Adults			
	HQ Ingestion	HQ Dermal	HQ Inhalation	HI	HQ Ingestion	HQ Dermal	HQ Inhalation	HI
Fe								
Minimum	3.54 × 10^−5^			3.54 × 10^−5^	4.04 × 10^−6^			4.04 × 10^−6^
Maximum	3.88 × 10^−4^			3.88 × 10^−4^	4.44 × 10^−5^			4.44 × 10^−5^
Mean	7.31 × 10^−5^			7.31 × 10^−5^	8.35 × 10^−6^			8.35 × 10^−6^
Co								
Minimum	2.49 × 10^−3^			2.49 × 10^−3^	2.85 × 10^−4^			2.85 × 10^−4^
Maximum	2.78 × 10^−2^			2.78 × 10^−2^	3.18 × 10^−3^			3.18 × 10^−3^
Mean	6.73 × 10^−3^			6.73 × 10^−3^	7.69 × 10^−4^			7.69 × 10^−4^
Cr								
Minimum	1.94 × 10^−2^	2.72 × 10^−3^	5.62 × 10^−5^	2.22 × 10^−2^	2.22 × 10^−3^	4.43 × 10^−4^	6.00 × 10^−5^	2.72 × 10^−3^
Maximum	6.16 × 10^−1^	8.63 × 10^−2^	1.78 × 10^−3^	7.05 × 10^−1^	7.05 × 10^−2^	1.41 × 10^−2^	1.90 × 10^−3^	8.64 × 10^−2^
Mean	4.46 × 10^−1^	6.25 × 10^−2^	1.29 × 10^−3^	5.10 × 10^−1^	5.10 × 10^−2^	1.02 × 10^−2^	1.38 × 10^−3^	6.26 × 10^−2^
Cu								
Minimum	4.45 × 10^−3^	4.15 × 10^−5^	1.23 × 10^−7^	4.49 × 10^−3^	5.09 × 10^−4^	6.76 × 10^−6^	1.31 × 10^−7^	5.15 × 10^−4^
Maximum	2.58 × 10^−2^	2.40 × 10^−4^	7.10 × 10^−7^	2.60 × 10^−2^	2.94 × 10^−3^	3.92 × 10^−5^	7.58 × 10^−7^	2.98 × 10^−3^
Mean	1.25 × 10^−2^	1.17 × 10^−4^	3.45 × 10^−7^	1.26 × 10^−2^	1.43 × 10^−3^	1.90 × 10^−5^	3.68 × 10^−7^	1.45 × 10^−3^
Ni								
Minimum	2.53 × 10^−3^	2.62 × 10^−5^	1.55 × 10^−5^	2.57 × 10^−3^	2.89 × 10^−4^	4.27 × 10^−6^	1.65 × 10^−5^	3.10 × 10^−4^
Maximum	5.83 × 10^−2^	6.05 × 10^−4^	3.57 × 10^−4^	5.93 × 10^−2^	6.66 × 10^−3^	9.85 × 10^−5^	3.81 × 10^−4^	7.14 × 10^−3^
Mean	2.09 × 10^−2^	2.17 × 10^−4^	1.28 × 10^−4^	2.12 × 10^−2^	2.39 × 10^−3^	3.53 × 10^−5^	1.37 × 10^−4^	2.56 × 10^−3^
Pb								
Minimum	1.27 × 10^−2^	2.38 × 10^−4^	3.49 × 10^−7^	1.30 × 10^−2^	1.46 × 10^−3^	3.88 × 10^−5^	3.72 × 10^−7^	1.49 × 10^−3^
Maximum	1.67 × 10^−1^	3.13 × 10^−3^	4.59 × 10^−6^	1.71 × 10^−1^	1.91 × 10^−2^	5.10 × 10^−4^	4.89 × 10^−6^	1.96 × 10^−2^
Mean	1.13 × 10^−1^	2.12 × 10^−3^	3.11 × 10^−6^	1.15 × 10^−1^	1.30 × 10^−2^	3.45 × 10^−4^	3.32 × 10^−6^	1.33 × 10^−2^
Zn								
Minimum	1.44 × 10^−3^	2.02 × 10^−5^	3.98 × 10^−8^	1.46 × 10^−3^	1.65 × 10^−4^	3.29 × 10^−6^	4.24 × 10^−8^	1.68 × 10^−4^
Maximum	1.40 × 10^−2^	1.96 × 10^−4^	3.86 × 10^−7^	1.42 × 10^−2^	1.60 × 10^−3^	3.19 × 10^−5^	4.12 × 10^−7^	1.63 × 10^−3^
Mean	4.10 × 10^−3^	5.75 × 10^−5^	1.13 × 10^−7^	4.16 × 10^−3^	4.69 × 10^−4^	9.36 × 10^−6^	1.21 × 10^−7^	4.79 × 10^−4^
Carcinogenic	CR ingestion	CR dermal	CR inhalation	TCR	CR ingestion	CR dermal	CR inhalation	TCR
Cr								
Minimum	2.50 × 10^−6^	2.8 × 10^−7^	5.79 × 10^−9^	2.79 × 10^−6^	1.43 × 10^−6^	2.28 × 10^−7^	3.09 × 10^−8^	1.69 × 10^−6^
Maximum	7.94 × 10^−5^	8.88 × 10^−6^	1.84 × 10^−7^	8.85 × 10^−5^	4.54 × 10^−5^	7.23 × 10^−6^	9.79 × 10^−7^	5.36 × 10^−5^
Mean	5.75 × 10^−5^	6.43 × 10^−6^	1.33 × 10^−7^	6.41 × 10^−5^	3.29 × 10^−5^	5.23 × 10^−6^	7.09 × 10^−7^	3.88 × 10^−5^
Ni								
Minimum	7.37 × 10^−6^	5.16 × 10^−7^	1.00 × 10^−10^	7.88 × 10^−6^	4.21 × 10^−6^	4.20 × 10^−7^	5.35 × 10^−10^	4.63 × 10^−6^
Maximum	1.70 × 10^−4^	1.19 × 10^−5^	2.31 × 10^−9^	1.82 × 10^−4^	9.71 × 10^−5^	9.68 × 10^−6^	1.23 × 10^−8^	1.07 × 10^−4^
Mean	6.09 × 10^−5^	4.26 × 10^−6^	8.29 × 10^−10^	6.51 × 10^−5^	3.48 × 10^−5^	3.47 × 10^−6^	4.42 × 10^−9^	3.83 × 10^−5^
Pb								
Minimum	3.25 × 10^−8^			3.2 × 10^−8^	1.86 × 10^−8^			1.86 × 10^−8^
Maximum	4.27 × 10^−7^			4.3 × 10^−7^	2.44 × 10^−7^			2.44 × 10^−7^
Mean	2.89 × 10^−7^			2.9 × 10^−7^	1.65 × 10^−7^			1.65 × 10^−7^

## Data Availability

The data can be found in the manuscript and supplementary file.

## Supplementary Materials

The following are available online at https://www.mdpi.com/article/10.3390/ijerph18168315/s1, Table S1: Instrument detection limits (DL) of trace elements determined by LA-ICP-MS, Table S2: Classes of EF and *Igeo*, Table S3: Definition and reference value of some parameters for health risk assessment of heavy metal in soils, Table S4: Reference doses (RfD) and slope factors (SF) for non-carcinogenic and carcinogenic trace metals, respectively, Table S5: Trace elements concentrations (in ppm) in the studied geophagic clays from Cameroon, Table S6: Rare earth elements concentrations (in ppm) in the studied geophagic clays from Cameroon.
