# Supplementary material for: Geophagic Clays from Cameroon: Provenance, Metal Contamination and Health Risk Assessment
Source: Int J Environ Res Public Health. 2021 Aug 5;18(16):8315. doi: 10.3390/ijerph18168315 (PMC8394028; doi:10.3390/ijerph18168315)
Supplement: Supplementary file 1 [file ijerph-18-08315-s001.zip › ijerph-1306260-supplementary.pdf]

Table S1: Instrument detection limits (DL) of trace elements determined by LA-ICP-MS.

Trace element	DL (ppm)	Trace element	DL (ppm)	Trace element	DL (ppm)
Sc	0.014	Mo	0.002	Ho	0.001
V	0.005	Cs	0.004	Er	0.001
Cr	0.093	Ba	0.004	Tm	0.001
Co	0.018	La	0.001	Yb	0.002
Ni	0.033	Ce	0.001	Lu	0.001
Cu	0.053	Pr	0.001	Hf	0.001
Zn	0.034	Nd	0.002	Ta	0.001
Rb	0.008	Sm	0.002	Pb	0.002
Sr	0.001	Eu	0.001	Th	0.001
Y	0.001	Gd	0.002	U	0.001
Zr	0.001	Tb	0		
Nb	0	Dy	0.001		

Table S2: Classes of EF and Igeo.

Index	Classes	Geophagic clay quality	References
EF	EF < 2	No or minimal enrichment	[1]
	2 < EF < 5	Moderate enrichment	
	5 < EF < 20	Significant enrichment	
	20 < EF < 40	Very high enrichment	
	EF > 40	Extremely enrichment	
I*geo*	Class 0: $I_{geo} < 0$	Uncontaminated	[2]
	Class 1: $0 < I_{geo} < 1$	Uncontaminated to moderately contaminated	
	Class 2: $1 < I_{geo} < 2$	Moderately contaminated	
	Class 3: $2 < I_{geo} < 3$	Moderately to heavily contaminated	
	Class 4: $3 < I_{geo} < 4$	Heavily contaminated	
	Class 5: $4 < I_{geo} < 5$	Heavily to extremely heavily contaminated	
	Class 6: $I_{geo} > 5$	Extremely contaminated	

Table S3: Definition and reference value of some parameters for health risk assessment of heavy metal in soils.

Parameters	Description	Units	Children	Adults	References
C_i	Trace metal concentration in soil	mg/kg			
CF	Conversion factor	kg/mg	10^{-6}	10^{-6}	[3]
EF	Exposure frequency	day/year	365	365	[4,5]
ED	Exposure duration	year	6	30	[5]
BW	Body weight	kg	16	70	[6]
AT_{nc}	Average time for non-carcinogenic effects	day	2190	10950	[7]
AT_{ca}^{b}	Average time for carcinogenic effects	day	-	25550	[4,5,7]
IR_{soil}	Ingestion rate	mg/day	200	100	[3]
IR_{air}	Respiratory rate	m^3/day	7.5	15	[8]
PEF	Particulate emission factor	m^3/kg	1.36×10^9	1.36×10^9	[5]
SA	Skin Surface Area	cm^2	2800	5700	[4,5]
AF	Adherence factor	mg/cm^2/day	0.2	0.07	[4,5]
ABS	Absorption factor	-	-	0.001	[4,5]

Table S4: Reference doses (RfD) and slope factors (SF) for non-carcinogenic and carcinogenic trace metals, respectively [4].

	Reference dose (RfD)			Slope factor (SF)		
	Ingestion	Inhalation	Dermal	Ingestion	Inhalation	Dermal
Co	2.00E-02	-	-	-	-	
Cr	3.00E−03	2.86E−05	6.00E−05*	5.01E−01	4.20E+01	2.00E+01
Cu	4.00E−02	4.00E−02	1.20E−02	-	-	–
Ni	2.00E−02	9.00E−05[1]	5.40E−03	1.70E+00	8.40E-01[1]	4.25E+01
Pb	3.50E−03*	3.52E−03	5.24E−04	8.50E−03	-	–
Fe	0.7	-	-	-	-	-
Zn	3.00E−01	0.3	6.00E−02	-	-	–

[1]Source: [9,10].

Table S5: Trace elements concentrations (in ppm) in studied geophagic clays from Cameroon

	Sc	V	Cr	Co	Ni	Cu	Rb	Sr	Y	Zr	Nb	Ba	Hf	Th	U	Ta	Pb	Mo	Cs	Zn
**Acacia**	24.51	142.80	115.12	26.93	83.87	76.24	104.53	107.45	59.33	571.39	54.60	1052.24	15.58	25.37	5.67	3.76	31.91	2.30	4.80	328.27
**Balengou**	12.30	7.69	5.28	11.94	8.12	35.82	2.24	2.82	105.94	1303.47	179.22	259.03	32.18	30.90	6.00	11.20	11.23	5.54	0.55	182.11
**Bokwango**	23.01	110.56	119.86	4.37	27.39	26.10	35.68	104.08	42.42	313.08	52.17	209.08	8.95	24.53	3.69	3.66	35.09	2.39	5.06	41.99
**Etoudi**	25.23	143.94	126.92	24.92	55.21	48.64	74.43	103.41	49.84	403.75	52.20	615.37	11.53	25.20	4.40	3.66	33.78	2.16	5.14	112.21
**Madagascar**	24.21	117.70	117.11	8.92	34.46	35.33	53.39	101.47	58.11	368.82	50.89	407.60	10.49	24.13	4.17	3.68	34.66	1.95	5.06	107.54
**Mendong**	23.79	101.35	122.00	5.55	24.51	37.38	36.07	108.18	43.70	326.65	52.73	219.15	9.45	22.61	3.43	3.82	37.01	1.84	4.99	42.97
**Mfoundi**	25.54	125.49	118.37	10.68	39.80	44.91	50.22	87.79	51.93	425.55	49.64	374.23	12.04	25.36	4.65	3.54	35.09	2.34	4.44	105.79
**Mokolo**	23.28	131.86	121.49	14.59	45.37	52.96	62.99	99.62	57.91	394.97	57.47	526.39	11.17	24.70	4.81	4.04	33.44	2.22	4.83	76.84
**Muda-Betsi**	22.87	148.35	121.03	30.33	81.34	53.24	101.57	102.16	61.20	471.68	55.37	967.23	13.07	24.15	5.46	3.82	33.08	4.46	4.55	276.27
**Mvo-Ada**	7.01	7.57	5.26	12.25	4.68	43.42	0.57	1.51	54.71	838.79	113.29	165.68	21.74	20.26	3.87	7.93	8.84	3.43	<0.111	202.59
**Mvog Ama**	27.89	123.57	144.76	6.16	23.73	22.80	34.51	115.20	45.84	302.59	51.27	225.70	8.78	24.79	4.08	3.74	41.27	2.21	4.77	41.54
**Mvog-Betsi**	19.90	95.79	98.74	5.97	19.69	37.94	24.40	57.79	50.59	678.67	80.07	140.43	18.39	26.94	4.65	5.47	24.04	3.01	4.05	57.68
**Nkol-Eton**	23.13	110.83	113.89	7.29	28.56	28.75	40.22	105.21	44.95	308.92	53.43	217.01	9.10	24.24	3.23	3.86	35.97	2.15	5.74	43.87
**Minimum**	7.01	7.57	5.26	4.37	4.68	22.80	0.57	1.51	42.42	302.59	49.64	140.43	8.78	20.26	3.23	3.54	8.84	1.84	0.55	41.54
**Maximum**	27.89	148.35	144.76	30.33	83.87	76.24	104.53	115.20	105.94	1303.47	179.22	1052.24	32.18	30.90	6.00	11.20	41.27	5.54	5.74	328.27
**Mean**	21.74	105.19	102.29	13.07	36.67	41.81	47.75	84.36	55.88	516.02	69.41	413.78	14.04	24.86	4.47	4.78	30.42	2.77	4.50	124.59

Table S6: Rare earth elements concentrations (in ppm) in studied geophagic clays from Cameroon

	La	Ce	Pr	Nd	Sm	Eu	Gd	Tb	Dy	Ho	Er	Tm	Yb	Lu
**Acacia**	95.54	180.35	23.05	84.66	15.54	3.00	13.51	1.94	11.50	2.23	6.47	0.91	6.18	0.88
**Balengou**	186.93	135.92	49.40	180.71	34.26	1.67	27.39	4.20	24.46	4.59	12.90	1.78	11.84	1.70
**Bokwango**	109.68	205.86	25.67	89.85	14.65	2.70	11.31	1.59	8.95	1.69	4.75	0.65	4.32	0.61
**Etoudi**	100.10	190.32	23.70	86.22	15.97	3.02	12.94	1.83	10.46	2.03	5.87	0.80	5.30	0.76
**Madagascar**	117.53	236.73	30.72	114.90	20.75	3.97	16.78	2.33	12.92	2.42	6.73	0.94	5.82	0.82
**Mendong**	108.58	203.34	25.93	92.09	15.08	2.82	11.77	1.65	9.30	1.75	4.93	0.73	4.61	0.68
**Mfoundi**	106.59	205.66	26.41	96.84	17.31	3.35	14.25	2.06	11.57	2.20	6.17	0.87	5.57	0.82
**Mokolo**	105.65	226.55	27.10	100.86	18.83	3.60	15.94	2.28	12.83	2.43	6.77	0.98	6.25	0.89
**Muda-Betsi**	102.32	193.79	25.43	94.00	17.09	3.48	14.86	2.15	12.20	2.37	6.94	0.99	6.22	0.95
**Mvo-Ada**	40.65	84.24	14.52	57.39	13.56	0.53	12.20	2.03	12.35	2.42	6.93	1.09	6.80	1.09
**Mvog Ama**	116.95	237.79	30.52	109.96	19.09	3.57	14.08	1.97	11.00	1.97	5.61	0.78	5.02	0.76
**Mvog-Betsi**	99.01	134.01	25.09	90.44	15.78	1.81	11.96	1.85	10.93	2.16	6.24	0.91	6.07	0.90
**Nkol-Eton**	108.72	195.73	24.83	89.33	15.55	2.98	12.66	1.76	9.98	1.87	5.28	0.76	4.77	0.70
**Minimum**	40.65	84.24	14.52	57.39	13.56	0.53	11.31	1.59	8.95	1.69	4.75	0.65	4.32	0.61
**Maximum**	186.93	237.79	49.40	180.71	34.26	3.97	27.39	4.20	24.46	4.59	12.90	1.78	11.84	1.70
**Mean**	107.56	186.94	27.10	99.02	17.96	2.81	14.59	2.12	12.19	2.32	6.58	0.94	6.06	0.89

References

1. Sutherland, R.A. Bed Sediment-Associated Trace Metals in an Urban Stream, Oahu, Hawaii. *Environmental Geology* **2000**, *39*, 611–627, doi:10.1007/s002540050473.
2. Muller, G. Index of Geoaccumulation in Sediments of the Rhine River. *Geojournal* **1969**, *2*, 108–118.
3. USEPA, (US Environmental Protection Agency) *Risk Assessment Guidance for Superfund*; DC: Office of Emergency and Remedial Response: Washington, USA, 1989;
4. Adimalla, N. Heavy Metals Contamination in Urban Surface Soils of Medak Province, India, and Its Risk Assessment and Spatial Distribution. *Environ Geochem Health* **2020**, *42*, 59–75, doi:10.1007/s10653-019-00270-1.
5. USEPA, (US Environmental Protection Agency) *Supplemental Guidance for Developing Soil Screening Levels for Superfund Sites*; Office of Emergency and Remedial Response: Washington, USA, 2002.
6. Li, F.; Zhang, J.; Cao, T.; Li, S.; Chen, Y.; Liang, X.; Zhao, X.; Chen, J. Human Health Risk Assessment of Toxic Elements in Farmland Topsoil with Source Identification in Jilin Province, China. *IJERPH* **2018**, *15*, 1040, doi:10.3390/ijerph15051040.
7. Gu, Y.-G.; Gao, Y.-P.; Lin, Q. Contamination, Bioaccessibility and Human Health Risk of Heavy Metals in Exposed-Lawn Soils from 28 Urban Parks in Southern China's Largest City, Guangzhou. *Applied Geochemistry* **2016**, *67*, 52–58, doi:10.1016/j.apgeochem.2016.02.004.
8. USEPA, (US Environmental Protection Agency) *Risk Assessment Guidance for Superfund*; DC: Office of Emergency and Remedial Response: Washington, USA, 2004.
9. Ferreira-Baptista, L.; De Miguel, E. Geochemistry and Risk Assessment of Street Dust in Luanda, Angola: A Tropical Urban Environment. *Atmospheric Environment* **2005**, *39*, 4501–4512, doi:10.1016/j.atmosenv.2005.03.026.
10. Li, Z.; Ma, Z.; van der Kuijp, T.J.; Yuan, Z.; Huang, L. A Review of Soil Heavy Metal Pollution from Mines in China: Pollution and Health Risk Assessment. *Science of The Total Environment* **2014**, *468–469*, 843–853, doi:10.1016/j.scitotenv.2013.08.090.
